# Red blood cell distribution width predicts coronary artery lesions in Kawasaki disease: insights from a Japanese cohort

**DOI:** 10.1186/s12969-025-01083-6

**Published:** 2025-03-25

**Authors:** Yamato Hanawa, Wataru Murasaki, Hiroyuki Namba, Kimihiko Oishi

**Affiliations:** 1 Department of Pediatrics, Jikei University Kashiwa Hospital, 163-1, Kashiwa-shita, Kashiwa, Chiba Japan; 2 Department of Pediatrics, Jikei University School of Medicine, 3-25-8, Nishi-Shimbashi, Minato-ku, Tokyo Japan

**Keywords:** Kawasaki disease, Red blood cell distribution width, Coronary artery lesion, Iron metabolism

## Abstract

**Background:**

Kawasaki disease (KD) is an acute vasculitis that causes coronary artery lesions. This study aimed to identify risk factors for the early prediction of coronary artery disease (CAD) in KD.

**Methods:**

We conducted a retrospective analysis of 175 Japanese children diagnosed with KD between January 2019 and March 2024. Univariate and multivariate logistic regression analyses were performed to identify predictors of CAD, and the diagnostic performance of various indicators was assessed using receiver operating characteristic (ROC) curves. The correlations between red blood cell distribution width (RDW) and iron-related anemia biomarkers were also evaluated.

**Results:**

Of these, 77 with CAD were classified into the CAD group, while 98 without CAD were categorized as the non-CAD group. Patients in the CAD group were younger and had lower levels of hemoglobin (Hb), total protein, albumin, uric acid, and urea nitrogen, but a higher RDW coefficient of variation (RDW-CV) than the non-CAD group. Logistic regression analysis identified RDW-CV as an independent predictor of CAD. ROC curve analysis demonstrated moderate predictive performance for RDW-CV, with an area under the curve of 0.636 (sensitivity, 55.8%; specificity, 70.4%). Significant correlations were observed between RDW-CV and iron-related anemia biomarkers in the CAD group, but not in the non-CAD group.

**Conclusions:**

Iron dysregulation may be associated with CAD, and RDW-CV may aid in identifying patients who may develop CAD in KD. Our findings were consistent with previous studies in other Asian populations, supporting the utility of RDW-CV as a predictor of CAD in KD in populations with various ethnic backgrounds.

## Introduction

Kawasaki disease (KD) is an acute febrile systemic vasculitis of unknown etiology primarily affecting the coronary arteries [1]. It is now recognized as the leading cause of acquired pediatric heart disease in children across developed countries [2]. Among its complications, coronary artery disease (CAD), such as dilation and aneurysms, present the most significant risk, potentially progressing to obstruction or stenosis, which may result in myocardial ischemia or even sudden death. Therefore, early identification of patients at risk of developing CAD in KD is crucial. Although previous studies have investigated various biomarkers associated with CAD in KD patients [3–5], consistent and reliable predictors remain limited, highlighting the need for further research.

Red blood cell distribution width (RDW) measures the variability of red blood cell (RBC) volume, expressed as either the standard deviation (SD) or the coefficient of variation percent (CV). This parameter is obtained by an automated hematology analyzer and calculated from the mean corpuscular volume. RDW is commonly used as a marker of iron-deficiency anemia (IDA) and has been proposed as a predictive biomarker for cardiovascular disease in adults [6, 7]. Recently, several studies have suggested that RDW may also be a valuable predictor of CAD in children during the acute phase of KD [8, 9]. Moreover, serum ferritin, a ubiquitous intracellular protein responsible for iron storage is widely used as an acute-phase reactant biomarker in clinical practice [10]. Elevated serum ferritin levels have also been strongly associated with CAD in patients with KD [11]. However, the relationship between RDW and iron regulation in KD patients has yet to be thoroughly explored and remains poorly understood.

This study aimed to evaluate the potential of RDW as a useful predictive marker for identifying patients at risk of developing CAD in KD. Additionally, we aimed to explore the relationship between iron regulation in KD by analyzing the correlations among RDW, ferritin, and hemoglobin (Hb) levels.

## Materials and methods

### Participants

This retrospective case-control study was conducted in accordance with the Declaration of Helsinki and was approved by the ethics committee of Jikei University (36–160). Data from patients with KD admitted to Jikei University Kashiwa Hospital between January 1, 2019, and March 31, 2024, were included in this study.

The inclusion criteria were: (1) patients younger than 15 years old; (2) diagnosis of complete KD according to the 2017 American Heart Association guidelines [12]; and (3) initial onset of KD. The exclusion criteria were: (1) incomplete clinical data; (2) recurrent KD; (3) incomplete KD; (4) prior intravenous immunoglobulin (IVIG) treatment at other medical institutions before admission or the absence of IVIG treatment during hospitalization; (5) pre-existing cardiac conditions, including congenital heart disease, cardiomyopathy, myocarditis, valvular heart disease, severe arrhythmia, or heart failure; and (6) hematologic disorders such as moderate-to-severe anemia, leukemia, and multiple myeloma, myelodysplastic syndrome.

### Echocardiography measurements

Experienced sonographers performed echocardiography during hospitalization. Patients with KD were diagnosed with CAD if they met one of the following criteria based on the previous studies [8, 9]: (1) a z-score of ≥ 2 in at least one of the following coronary arteries—the right, left anterior descending, or left main—calculated using the Kobayashi z-score system [13]; or (2) an internal lumen diameter of > 2.5 mm in patients younger than three years old, > 3 mm in patients aged 3–9 years, or > 3.5 mm in patients aged 9–14 years.

### Group assignment

After applying the inclusion and exclusion criteria, patients were categorized into either the CAD (KD-CAD) or the non-CAD (KD-nCAD) groups based on their echocardiographic findings.

### Data collection

Demographic characteristics, laboratory data, and echocardiographic data were collected from the medical records. Laboratory data included white blood cell (WBC) count, neutrophil count, lymphocyte count, platelet (PLT) count, platelet distribution width (PDW), RBC count, Hb, C-reactive protein (CRP), ferritin, aspartate aminotransferase (AST), alanine aminotransferase (ALT), total bilirubin (T-Bil), direct bilirubin (D-Bil), total protein (TP), albumin (Alb), uric acid (UA), urea nitrogen (UN), lactate dehydrogenase (LDH), creatine kinase (CK), serum sodium (Na), serum potassium (K), serum chloride (Cl), fibrinogen (Fbg), prothrombin time international normalized ratio (PT-INR), activated partial thromboplastin time (APTT), and D-dimer. Venous blood samples were collected within 24 h before IVIG treatment. RDW-CV and RDW-SD were measured using the XE-5000 (Sysmex, Kobe, Japan), an automated hematology analyzer.

### Statistical analysis

The Shapiro–Wilk test was used to assess the distribution of variables. Continuous variables are presented as mean ± SD and were compared between the two groups using unpaired two-tailed t-tests. Data with non-normal distributions were presented as median (interquartile range) and compared between the two groups using the Mann–Whitney U test. Qualitative data were expressed as numbers and percentages and compared using χ2 tests. Univariate and multivariate logistic regression analyses were performed to identify independent predictors of CAD. The area under the receiver operating characteristic (ROC) curve (AUC) was analyzed to assess the predictive accuracy of the indicators for CAD and to determine the optimal cut-off point.

Pearson’s correlation was used to analyze the relationship between RDW and Hb. The correlation analysis between RDW and serum ferritin was conducted using Spearman’s correlation, as the relationship between these variables was non-linear.

All *p*-values were two-sided; values of 0.05 or less were considered statistically significant. All statistical analyses were performed using EZR (Saitama Medical Center, Jichi Medical University, Saitama, Japan) [14], which is a graphical user interface for R (The R Foundation for Statistical Computing, Vienna, Austria, version 4.3.1). Specifically, EZR is a modified version of R Commander (version 1.6–8) designed to include statistical functions commonly used in biostatistics.

## Results

### Analysis of baseline characteristics and laboratory findings

A total of 243 patients with KD were included in this study, and after applying the exclusion criteria, 175 patients remained for analysis, consisting of 111 males and 64 females, aged between 1 and 103 months. These patients have no history of iron, Vitamin B12, or folate supplementation. Based on echocardiographic findings, 77 patients (44%) were assigned to the CAD group, and 98 patients (56%) were assigned to the non-CAD group. The demographics and baseline laboratory findings are summarized in Table 1. There were no significant differences in sex, WBC, neutrophil count (N), lymphocyte count (L), ferritin, PDW, RDW-SD, RBC, PLT, Fbg, T-Bil, D-Bil, CK, LDH, Na, K, Cl, and CRP between the two groups (all *p* > 0.05). Age, and the levels of Hb, TP, Alb, UA, and UN in the CAD group, were significantly lower than those in the non-CAD group (all *p* < 0.05). Additionally, children in the CAD group had higher RDW-CV levels than those in the non-CAD group (*p* < 0.05).

**Table 1:** Demographic and laboratory characteristics of patients with Kawasaki disease in Jikei University Kashiwa Hospital between Jan 2019-March 2024 in this study

Variables	All (*n*=175)	CAD (*n*=77)	n-CAD (*n*=98)	*p* value
Age (month), mean ± SD	27.8 ± 19.3	22.9 ± 18.5	31.7 ± 19.1	0.003
Male (n, %)	111, 63.4	49, 63.6	62, 63.3	1
WBC (×10^9^/L), median (IQR)	13.4 (10.6, 16.4)	13.6 (10.5, 16.6)	13.5 (10.8, 16.2)	0.9
N (×10^9^/L), median (IQR)	8.8 (6.4, 12.0)	7.9 (6.5, 10.7)	9.4 (6.2, 12.1)	0.4
L (×10^9^/L), mean ± SD	3.5 ± 1.8	3.8 ± 2.0	3.3 ± 1.6	0.09
RDW-CV (%), mean ± SD	13.6 ± 1.1	13.8 ± 1.1	13.3 ± 0.9	0.002
RDW-SD (fL), mean ± SD	39.0 ± 2.9	39.5 ± 2.9	38.7 ± 2.9	0.06
PLT (×10^9^/L), median (IQR)	347 (272.5, 419.5)	351 (292, 446)	341 (268, 401.8)	0.1
PDW (%), mean ± SD	10.0 ± 1.1	10.1 ± 1.0	9.9 ± 1.2	0.4
RBC (×10^12^/L), mean ± SD	4.3 ± 0.5	4.3 ± 0.5	4.4 ± 0.5	0.1
Hb (g/dL), mean ± SD	11.4 ± 1.4	11.1 ± 1.3	11.7 ± 1.3	0.003
CRP (mg/dL), mean ± SD	7.5 ± 5.1	7.6 ± 5.5	7.4 ± 4.7	0.8
Ferritin (ng/mL), median (IQR)	123 (82, 174)	123 (79, 176)	124 (87.3, 172)	0.9
AST (U/L), median (IQR)	33 (25, 49.5)	32 (24, 52)	33 (25, 45.8)	0.6
ALT (U/L), median (IQR)	21 (13, 50.5)	29 (15, 49)	19 (12, 52.8)	0.2
T-Bil (mg/dL), median (IQR)	0.4 (0.3, 0.5)	0.4 (0.3, 0.6)	0.4 (0.3, 0.5)	0.5
D-Bil (mg/dL), median (IQR)	0.2 (0.1, 0.2)	0.1 (0.1, 0.2)	0.2 (0.1, 0.2)	0.6
TP (g/dL), mean ± SD	6.3 ± 0.6	6.1 ± 0.6	6.5 ± 0.6	<0.001
Alb (g/dL), mean ± SD	3.5 ± 0.4	3.3 ± 0.4	3.5 ± 0.4	0.008
UA (mg/dL), mean ± SD	3.7 ± 1.4	3.4 ± 1.4	3.9 ± 1.3	0.02
UN (mg/dL), mean ± SD	7.8 ± 3.2	7.0 ± 3.1	8.3 ± 3.0	0.006
LDH (U/L), median (IQR)	298 (266.5, 339)	309 (262, 348)	292 (267, 331.5)	0.5
CK (IU/L), median (IQR)	57 (41, 81)	57 (38, 84)	57.5 (41.3, 79.8)	0.7
Na (mmol/L), mean ± SD	134.5 ± 3.2	134.4 ± 3.4	134.5 ± 3.0	0.8
K (mmol/L), mean ± SD	4.3 ± 0.6	4.3 ± 0.6	4.3 ± 0.6	0.8
Cl (mmol/L), mean ± SD	100.8 ± 3.2	101.3 ± 3.5	100.5 ± 2.9	0.1
Fbg (mg/dL), median (IQR)	644 (552, 778)	619 (557, 749)	662 (551, 809)	0.1
PT-INR, mean ± SD	1.1 ± 0.1	1.1 ± 0.1	1.1 ± 0.1	0.4
APTT (s), median (IQR)	32.4 (29.9, 35.3)	32.4 (30.6, 34.9)	32.2 (29.7, 35.9)	0.8
D-dimer (µg/mL), mean ± SD	2.1 ± 1.3	2.2 ± 1.5	2.0 ± 1.2	0.3

*CAD* Coronary artery disease, *WBC* White blood cell, *N* Neutrophil count, *L* Lymphocyte count, *RDW* Red blood cell distribution width, *SD* Standard deviation, *CV* Coefficient of variation percent, *PLT* Platelet, *PDW* Platelet distribution width, *RBC* Red blood cell, *Hb* Hemoglobin, *CRP* C-reactive protein, *AST* Aspartate aminotransferase, *ALT* Alanine aminotransferase, *T-Bil* Total bilirubin, *D-Bil* Direct bilirubin, *TP* Total protein, *Alb* Albumin, *UA* Uric acid, *UN* Urea nitrogen, *LDH* Lactate dehydrogenase, *CK* Creatine kinase, *Na* Sodium, *K* Potassium, *Cl* Chloride, *Fbg* Fibrinogen, *PT-INR* Prothrombin time international normalized ratio *APTT* Activated partial thromboplastin time

### Independent risk factors for predicting CAD by logistic regression analysis

We performed univariate and multivariate logistic regression analyses on the parameters that demonstrated statistically significant differences between the CAD and non-CAD groups (Table 2). The univariate logistic regression analysis showed that age (OR = 1.0, 95% CI: 0.6–0.7, *p* = 0.003), RDW-CV (OR = 1.6, 95% CI: 0.6–0.7, *p* = 0.003), Hb (OR = 0.7, 95% CI: 0.5–0.7, *p* = 0.005), TP (OR = 0.4, 95% CI: 0.6–0.8, *p* < 0.001), Alb (OR = 0.3, 95% CI: 0.6–0.7, *p* = 0.001), UA (OR = 0.8, 95% CI: 0.5–0.7, *p* = 0.002), and UN (OR = 0.9, 95% CI: 0.5–0.7, *p* = 0.007) were significantly associated with CAD in patients with KD. Multivariate logistic regression analysis identified RDW-CV (OR = 1.5, 95% CI: 1.1–2.1, *p* = 0.02) as an independent predictor of CAD in patients with KD.

**Table 2:** Univariate and multivariate logistic regression analysis of independent factors for CAD in patients with Kawasaki disease

Independent factors	Univariate analysis		Multivariate analysis	
	OR (95%CI)	*p* value	OR (95%CI)	*p* value
Age (month)	0.97 (0.6-0.7)	0.004	0.98 (0.96-1.0)	0.07
RDW-CV (%)	1.6 (0.6-0.7)	0.003	1.5 (1.0-2.1)	0.02
Hb (g/dL)	0.69 (0.5-0.7)	0.005	0.94 (0.7-1.3)	0.7
TP (g/dL)	0.36 (0.6-0.8)	<0.001	0.66 (0.3-1.6)	0.4
Alb (g/dL)	0.27 (0.6-0.7)	0.001	0.39 (0.1-1.4)	0.2
UA (mg/dL)	0.75 (0.5-0.7)	0.02	0.87 (0.7-1.2)	0.3
UN (mg/dL)	0.87 (0.5-0.7)	0.007	1.0 (0.9-1.1)	0.8

*OR *Odds ratio, *CI *Confidence interval, *RDW *Red blood cell distribution width, *CV *Coefficient of variation percent, *Hb *Hemoglobin, *TP *Total protein, *Alb *Albumin, *UA *Uric acid, *UN *Urea nitrogen

### ROC analysis

**Fig. 1:**
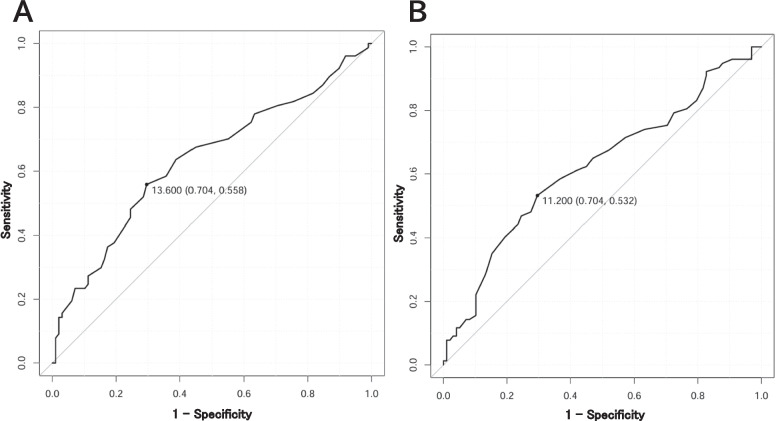
**A** ROC of RDW-CV for predicting CAD in patients with Kawasaki disease in Jikei University Kashiwa Hospital between Jan 2019-March 2024 in this study. **B** ROC of Hb for predicting CAD in patients with Kawasaki disease in Jikei University Kashiwa Hospital between Jan 2019-March 2024 in this study

The ROC curves for RDW-CV and Hb as predictors of CAD in KD patients were analyzed (Fig. 1). The ROC curve analysis identified the optimal cut-off value for RDW-CV as > 13.6%, with an AUC of 0.636, a sensitivity of 55.8%, and a specificity of 70.4%. The cut-off value for Hb was determined to be < 11.2 g/dL, yielding an AUC value of 0.623, with a sensitivity of 53.2% and a specificity of 70.4%, respectively. Table 3 shows the area under ROC curves for independent risk factors of CAD in KD patients.

**Table 3:** Area under the ROC curve and the optimal cut-off values for independent risk factors of CAD in patients with Kawasaki disease

Index	AUC	Best cut-off	Sensitivity (%)	Specificity (%)
Age (month)	0.651	≤21	62.3	66.3
RDW-CV (%)	0.636	≥13.6	55.8	70.4
Hb (g/dL)	0.623	≤11.2	53.2	70.4
TP (g/dL)	0.674	≤6	50.6	80.6
Alb (g/dL)	0.637	≤3.5	68.8	52.0
UA (g/dL)	0.624	≤3.2	54.5	69.4
UN (g/dL)	0.615	≤4.0	29.9	88.8

*AUC *Area under the receiver operating characteristic curve, *RDW *Red blood cell distribution width, *CV *Coefficient of variation percent, *Hb *Hemoglobin, *TP *Total protein, *Alb *Albumin, *UA *Uric acid, *UN *Urea nitrogen

### Correlation analysis

**Fig. 2:**
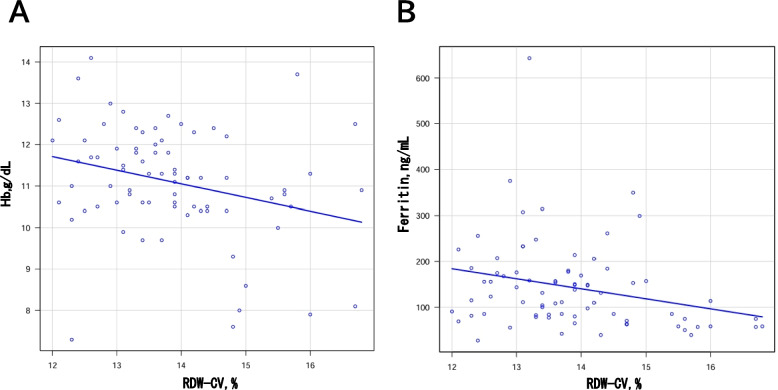
**A** Scatter plot of RDW-CV and Hb values of the CAD group in this study. Pearson correlation coefficient = - 0.3; *p *= 0.01. **B** Scatter plot of RDW-CV and serum ferritin values of the CAD group in this study. Spearman correlation coefficient = -0.3; *p *= 0.005

**Fig. 3:**
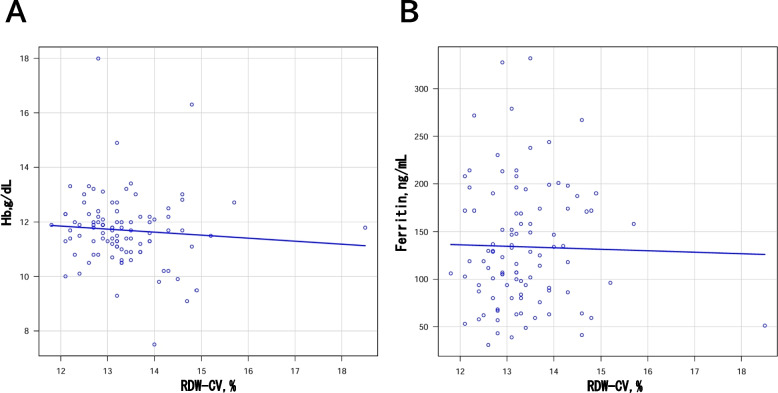
**A** Scatter plot of RDW-CV and Hb in the non-CAD group in this study. Pearson correlation coefficient = - 0.08; *p *= 0.4. **B** Scatter plot of RDW-CV and serum ferritin in the non-CAD group in this study. Spearman correlation coefficient = 0.05; *p *= 0.6

We conducted a correlation analysis to further investigate the potential relationship between RDW-CV and iron regulation in KD. This analysis aimed to determine whether RDW-CV, along with other iron-related parameters, could provide additional insights into the development of CAD in KD patients. Scatter plots were generated to examine the correlation between RDW-CV and Hb, as well as RDW-CV and serum ferritin (Figs. 2 and 3). A significant negative correlation was observed between RDW-CV and Hb (*r* = −0.3, *p* = 0.01), and between RDW-CV and serum ferritin (*r* = −0.3, *p* = 0.005) in the CAD group. In contrast, no significant correlation was observed between RDW-CV and Hb (*r* = −0.08, *p* = 0.4), or between RDW-CV and serum ferritin (*r* = 0.05, *p* = 0.6) in the non-CAD group.

## Discussion

This retrospective study evaluated the utility of laboratory parameters, particularly RDW-CV, in predicting CAD in patients with KD. Our findings suggest that RDW-CV may serve as an independent predictor for CAD in KD, with significant correlations observed between RDW-CV and anemia-related biomarkers in the CAD group. Although RDW-CV emerged as a potential predictive marker, it is important to acknowledge that the identified cut-off value (> 13.6%) falls within the normal reference range, which is generally considered to be up to 14.5% in healthy individuals. However, RDW-CV in patients with KD may be influenced by systemic inflammation, leading to shifts within the normal range that still hold clinical significance. In this context, the threshold of 13.6% identified in our study may better reflect disease-specific alterations rather than general population norms. While this value may still offer clinical utility in identifying patients at higher risk for CAD, the slight difference compared to the non-CAD group limits its practical application at this time. This narrow margin could reduce its sensitivity in distinguishing patients at risk. Therefore, RDW-CV should be viewed as a potentially helpful marker but not sufficient for robust risk stratification. Instead, it should be incorporated into a multimodal approach alongside other hematological and biochemical parameters to improve diagnostic accuracy. While the sensitivity (55.8%) is moderate, the specificity (70.4%) suggests that RDW-CV may still be useful in ruling out certain conditions when used in conjunction with other markers.

Our findings align with previous reports from other Asian populations, further supporting the utility of RDW-CV as a predictor of CAD in KD [8, 9, 15–17]. However, this is the first study to identify RDW-CV as a predictive marker, specifically in the Japanese population. This reinforces the relevance of RDW-CV as a biomarker in the context of KD, particularly among Asian populations, and suggests that its utility may extend across different ethnic groups.

RDW is known to reflect variations in RBC size, typically associated with IDA [18]. However, RDW can increase beyond IDA due to inflammation and oxidative stress, both of which can disrupt RBC homeostasis. Elevated RDW has been proposed as a new inflammatory biomarker in various conditions, including cardiovascular disease [19]. Several previous studies have already linked higher RDW levels with the development of CAD in patients with KD [8, 9, 15–17], and our findings are consistent with these findings.

Regarding iron metabolism, ferritin, a well-established marker of inflammation and iron storage, showed a strong association with CAD [11]. This may be linked to the role of hepcidin, an inflammation-driven molecule that modulates iron homeostasis by reducing serum iron levels, leading to impaired erythropoiesis and elevated RDW [20, 21]. While we did not measure hepcidin levels as a routine test item, our findings of a significant negative correlation between RDW and both Hb and serum ferritin in the CAD group suggest that dysregulation of iron metabolism may contribute to the pathophysiological mechanism of CAD in KD patients. The absence of these correlations in the non-CAD group further highlights the potential role of disrupted iron metabolism in CAD development. This suggests that inflammatory and autoimmune responses may interfere with iron regulation, thus influencing RDW-CV and CAD risk.

Our study found a non-significant negative correlation between RDW-CV and ferritin in the non-CAD group. Ferritin is a major iron storage protein, but it also functions as an acute-phase reactant. Iron utilization is restricted in the presence of inflammation, and ferritin levels increase [10]. Therefore, the inflammatory response in our study may have influenced ferritin levels, making it difficult to detect a simple negative correlation with RDW-CV.

However, it is important to recognize our study’s limitations. (1) The study’s retrospective design and single-center nature limit our findings’ generalizability. (2) The relatively small sample size may reduce the statistical power and introduce bias into the analyses. (3) Several sonographers performed echocardiography, and differences in skill should be taken into consideration. (4) We did not account for other potential confounding variables, such as the influence of treatment modalities or other coronary artery lesions, which could have impacted our results. (5) Moreover, our study population was exclusively Asian, and including non-Asian populations would enhance the generalizability of our findings. Further large-scale-prospective multicenter studies are required to validate the role of RDW-CV in predicting CAD and to explore the mechanistic links between iron metabolism and KD more thoroughly.

## Conclusion

Our study suggests that RDW-CV may be a useful marker for predicting CAD in KD patients. The observed association between RDW-CV and markers of iron metabolism, particularly in the CAD group, indicates that iron dysregulation may be a contributing factor in the pathophysiology of CAD. The consistency of our findings with previous studies in other Asian populations reinforces the utility of RDW-CV, especially given that this is the first time it has been identified as a predictor in the Japanese population. Nonetheless, further research is needed to fully understand the clinical utility of RDW-CV and the complex interplay between iron metabolism, inflammation, and autoimmune responses in KD.

## Data Availability

Data of this work are available upon request.

## Abbreviations

Alb: Albumin
ALT: Alanine aminotransferase
APTT: Activated patrial thromboplastin time
AST: Aspartate aminotransferase
AUC: Area under ROC curve
CAD: Coronary artery disease
CI: Confidence interval
CK: Creatine kinase
Cl: Serum chloride
CRP: C-reactive protein
CV: Coefficient of variation
D-Bil: Direct bilirubin
Fbg: Fibrinogen
Hb: Hemoglobin
IVIG: Intravenous immunoglobulin
K: Serum potassium
KD: Kawasaki disease
L: Lymphocyte count
LDH: Lactate dehydrogenase
N: Neutrophil count
Na: Serum sodium
OR: Odds ratio
PDW: Platelet distribution width
PLT: Platelet
PT-INR: Prothrombin time international normalized ratio
RBC: Red blood cell
RDW: Red blood cell distribution width
ROC: Receiver operating characteristic
SD: Standard deviation
T-Bil: Total bilirubin
TP: Total protein
UA: Uric acid
UN: Urea nitrogen
WBC: White blood cell
